# Essential Oil Composition and Stable Isotope Profile of Cultivated *Ocimum campechianum* Mill. (Lamiaceae) from Peru

**DOI:** 10.3390/molecules27092777

**Published:** 2022-04-27

**Authors:** Tyler M. Wilson, Brett J. Murphy, Adrian Abad, Chris Packer, Ariel Poulson, Richard E. Carlson

**Affiliations:** D. Gary Young Research Institute, Lehi, UT 84043, USA; bmurphy@youngliving.com (B.J.M.); adabad@youngliving.com (A.A.); cpacker@youngliving.com (C.P.); apoulson@youngliving.com (A.P.); richcarlson@youngliving.com (R.E.C.)

**Keywords:** chemotype, essential oil, *Ocimum campechianum*, Peruvian basil, stable isotope, yield

## Abstract

*Ocimum campechianum* Mill. (Peruvian basil) is an essential oil-bearing plant of the Lamiaceae family. Volatile oil produced through steam distillation of Peruvian basil was examined to establish the aromatic and stable isotope profiles of samples (*n* = 9) from three different cultivated plots in Peru. The resulting essential oils were analyzed by GC/FID, GC/MS, and GC/IRMS. In accordance with findings from other researchers, multiple chemotypes, defined by the most abundant aromatic compounds, exist within these populations. Overall, 55% of samples are the eugenol chemotype (values ranging 15.4–30.2%), 33% are the methyl eugenol chemotype (values ranging 68.1–68.7%), and a single sample is a mixture of both chemotypes, containing high levels of both eugenol (38.1%) and methyl eugenol (8.6%). Stable isotope ratios, *δ*^2^H and *δ*^13^C, performed on prominent compounds provide supporting data for distinguishing chemotypes. Complete aromatic profiles, stable isotope ratios, and essential oil yield are established for each sample. This study confirms the existence of multiple chemotypes and, for the first time, to the author’s best knowledge, establishes stable isotope ratios for *O. campechianum* essential oil, which proves a useful tool in further investigating plant metabolism and determining essential oil authenticity.

## 1. Introduction

*Ocimum campechianum* Mill. (syn. *Ocimum micranthum* Willd.; common name Peruvian basil), is an essential oil-bearing plant of the Lamiaceae family that is native to the Americas, including a wide geographic region in North and South America and several islands in the Caribbean islands [1,2]. Given such a large geographic origin, Peruvian basil is known by many common names, including wild Amazonian basil, albahaca de monte, albahaca de las tierra, and albahaca silvestre [3,4,5]. The flowering plant, which grows to 1 m in height, has aromatic leaves that contain two types of glandular trichomes, peltate and capitate, from which essential oil can be extracted [2,6,7]. Other secondary metabolites have also been isolated from the leaves, including non-volatile terpenoids, polyphenols, tannins, and flavonoids [8,9,10].

**Figure 1:**
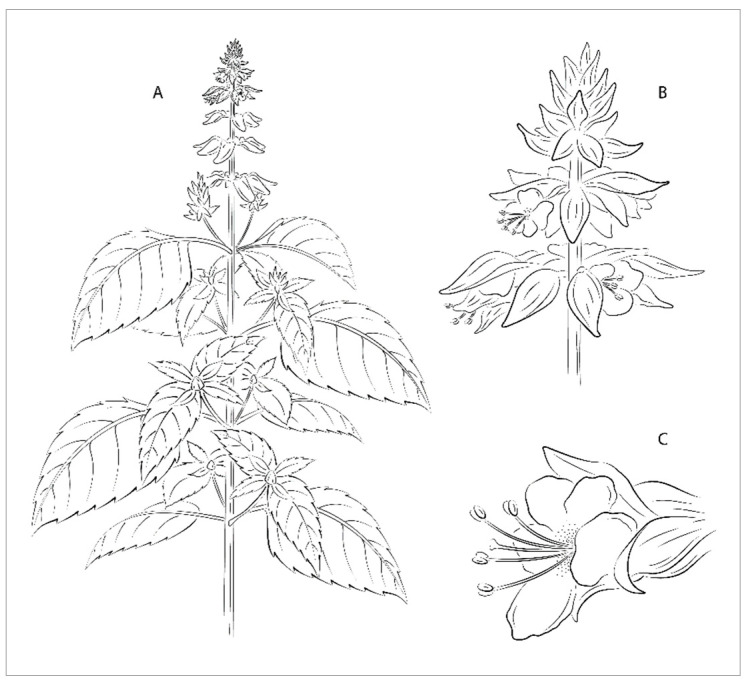
Botanical illustration of *Ocimum campechianum* Mill. showing (**A**) inflorescence and multiple sets of leaves, (**B**) inflorescence, and (**C**) single flowering structure. Illustrated by Rick Simonson, Science Lab Studios, Inc. (Kearney, NE, USA).

Many morphological parts of the Peruvian basil plant have associated ethnobotanical significance, with use of the leaves as the most common practice. In some areas, this plant is viewed as a valued and prized remedy [11] and/or used for culinary purposes [1,2,6]. Among over 50 traditional uses for the Peruvian basil plant (Figure 1), those most commonly cited are the purported anti-inflammatory, antirheumatic, and anticonvulsive properties [2,4,11]. Leaf decoctions, salves, and other plant applications are commonly used to treat aches, pain, sores, bronchitis, cold, cough, fever, flu, diarrhea, dysentery, and hypertension [2,4,12,13,14]. Women are reported to use the plant as an emmenagogue, to aid in childbirth, and to treat colic children [2,4,12].

Many of these traditional uses have been substantiated through recent studies. While most studies reviewed here did not show strong antibacterial properties of Peruvian basil essential oil [3,15,16], all showed strong antifungal properties in both the extracted essential oil [9,17,18] and in the plant during cultivation [19]. Peruvian basil extracts have demonstrated antiproliferative [15], antinociceptive [20], antiradical [3], antioxidant [3,9,21], anesthetic [22], and hypotensive [23] properties in various studies and models. Extracts have also demonstrated phytotoxic [24], insect repellant [25], and larvicidal [26] properties, suggesting a potential application as a natural herbicide and/or pesticide. The number of substantiated applications for Peruvian basil essential oil and extracts appears to be overshadowed by the number and diversity of ethnobotanical applications. 

The active properties of Peruvian basil essential oil are contingent on the aromatic profile. Multiple chemotypes, defined by the most abundant aromatic compounds in the essential oil, exist within this species including those with prominent aromatic compounds being methyl eugenol [17,25], 1,8-cineole [16,27], methyl-*E*-cinnamate [28], (*E*)-caryophyllene [27], methyl chavicol [22], or eugenol [3,9,18,21,24,26,29,30]. However, the term ‘chemotype’ may be somewhat plastic as the aromatic profile is strongly influenced by the portion of the plant used in the extraction process [17,29,30], seasonal variation [17], and plant development and maturity [31]. 

This study confirms the existence of multiple chemotypes within cultivated populations in Peru and, for the first time, to the author’s best knowledge, establishes stable isotope ratios for *O. campechianum* essential oil. Distillation yields are also reported. Results provide further insight into chemical variation and provide fundamental data for continual substantiation of ethnobotanical applications. 

## 2. Results

Consistent with previous findings [3,9,17,18,21,24,25,26,29,30], multiple chemotypes exist within the plant species. The three samples from the Pueblo Libre Region (samples A–C) conform to the methyl eugenol profile chemotype and the samples from the El Diamante (samples D–F) and Villa Rica (samples G, I) regions conform to the eugenol profile chemotype, with the exception of sample H, which contains a high relative area % of both eugenol (38.1%) and methyl eugenol (8.6%). The aromatic profile of each sample is detailed in Table 1.

Stable isotope ratios, *δ*^2^H and *δ*^13^C, were established for prominent compounds in essential oil samples. Prominent compounds are defined by the relative area % of a given compound being >5% for all samples from a region, including 1,8-cineole (samples D–I), cis-β-ocimene (samples D–I), eugenol (samples D–I), methyl eugenol (samples A–C), and (E)-caryophyllene (samples A–I). Stable isotope data is detailed in Table 2. 

Essential oil yield is detailed in Table 3. The average yields per collection region are 2.9 mL/kg (Pueblo Libre), 2.5 mL/kg (El Diamante), and 2.4 mL/kg (Villa Rica). 

## 3. Discussion

This study provides the essential oil profiles and, for the first time, to the author’s best knowledge, establishes stable isotope ratios for *Ocimum campechianum* essential oil. According to the aromatic profiles, two chemotypes exist within these samples (*n* = 9). The three samples from the Pueblo Libre Region (samples A–C) conform to the methyl eugenol profile chemotype, the samples from the El Diamante (samples D–F) and Villa Rica (samples G, I) regions conform to the eugenol profile chemotype, and sample H, which contains a high relative area % of both eugenol (38.1%) and methyl eugenol (8.6%), could be considered a mixture of the two chemotypes. In the scientific literature previously reviewed, Peruvian basil chemotypes included both the eugenol [3,9,18,21,24,26,29,30] and methyl eugenol [17,25] chemotypes. In the eugenol chemotypes referenced, methyl eugenol was not detected in any sample studied with the exception of one [30]. However, this same study distinguished the essential oil profiles from the leaf and inflorescence of Peruvian basil, where eugenol (44.8%, 14.0%) and methyl eugenol (1.7%, 6.2%) were prominent compounds detected, respective to each plant part. In the current study, the prominence of methyl eugenol (8.6%), extracted from the leaves and inflorescence of sample H, stands apart from all other eugenol chemotype essential oils previously studied and is likely a mixture the two chemotypes rather than a third, and distinct, chemotype. 

**Figure 2:**
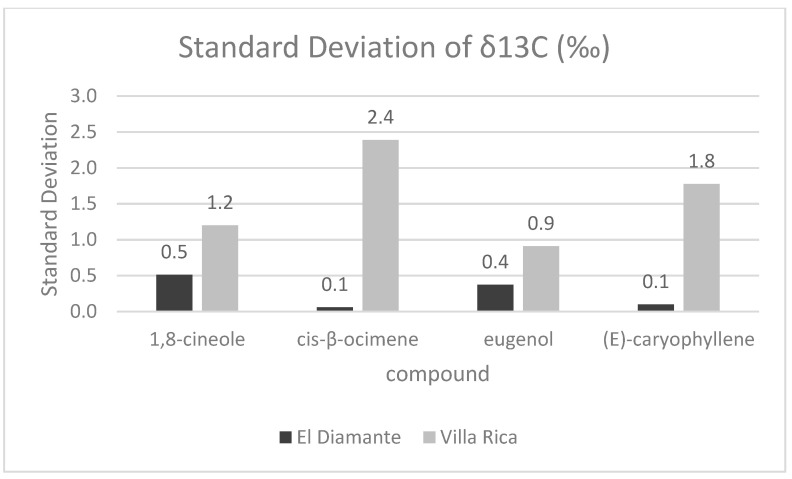
Standard deviations of *δ*^13^C values for 1,8-cineole, *cis*-β-ocimene, eugenol, and (*E*)-caryophyllene for two regions, El Diamante and Villa Rica.

The ability to distinguish sample H from the other samples analyzed is further supported by stable isotope ratio data. While conventional taxonomy and plant identification maintain chemotypes as indistinguishable, the stable isotope analysis provides insight into the molecular workings of the plant. The *δ*^13^C results of all four compounds analyzed from sample H are outliers within the Villa Rica group. As a result, the standard deviation for *δ*^13^C according to collection area is up to 24× larger in the Villa Rica compared to the El Diamante collection areas (Figure 2).

The stable isotope data for (*E*)-caryophyllene is the only data measured for all 9 samples, due to the abundance of this compound in each sample. (*E*)-caryophyllene *δ*^13^C ranges for the Pueblo Libre collection area are −32.850‰ to −32.688‰ and the ranges for the El Diamante and Villa Rica collection areas, excluding sample H, are −29.838‰ to −28.059‰. The (*E*)-caryophyllene *δ*^13^C value for sample H sits in between each range, at −31.612‰. These data provide further support that stable isotope ratios may be a useful tool in chemotyping by providing additional insight into the metabolism and carbon sequestration of the plant. 

This study provides further insight into chemical variation of *O. campechianum* essential oil and provides fundamental data for continual substantiation of ethnobotanical applications. Additional research is needed to investigate chemotypes within other regions where *O. campechianum* is found and to investigate the utility of stable isotope analysis as a chemotyping tool.

## 4. Materials and Methods

Essential oil samples were collected from distilleries in 3 producing regions in Peru (*n* = 3 (per distillery) × 3 (distilleries) = 9 (total samples)). Plant material was collected and distilled during the first and second week of January 2022. Plant material was collected within a 200 m radius of the following three locations: Pueblo Libre: 5°52′46.8″ S 77°7′27.6″ W, 830 m elevation; El Diamante: 5°44′19.9″ S 77°30′0.2″ W, 950 m elevation; Villa Rica: 5°47′2.7″ S 77°28′52.3″ W, 935 m elevation. Collected plant material is representative of the above ground portions of the plant, primarily consisting of the aromatic leaves and inflorescence (Figure 1). For simplicity and consistency, each sample is referred to by a letter, A-I (Table 4). Representative voucher samples from the three locations are held in the herbarium located at the Universidad Nacional de Cajamarca (Herbario Isidoro Sánchez Vega_UNC; herbarium code CPUN): Campos 2022-01, -02, -03 (CPUN).

For steam distillation, plant material was accurately weighed and added to a 125 L stainless steel distillation chamber, distilled for 2 h from pass-over by direct steam, essential oil was separated by a cooled condenser and Florentine flask, and accurately measured for yield. Essential oil samples were each filtered by centrifugation and stored at room temperature in a sealed amber bottle until analysis. 

Essential oils were analyzed, and volatile compounds identified, by GC/MS using an Agilent 7890B GC/5977B MSD (Agilent Technologies, Santa Clara, CA, USA) and Agilent J&W DB-5, 60 m × 0.25 mm, 0.25 μm film thickness, fused silica capillary column. Operating conditions: 0.1 μL of sample (20% soln. for essential oils in ethanol), 150:1 split ratio, initial oven temperature of 40 °C with an initial hold time of 5 min, oven ramp rate of 4.5 °C per minute to 310 °C with a hold time of 5 min, helium carrier gas. The electron ionization energy was 70 eV, scan range 35–650 amu, scan rate 2.4 scans per second, source temperature 230 °C, and quadrupole temperature 150 °C. Volatile compounds were identified using the Adams volatile oil library [32] using Chemstation library search in conjunction with retention indices. Note that 1-octen-3-ol/β-pinene and α-*trans*-bergamotene/γ-elemene elute as unresolved peaks. Their ratios were determined by the ratio of masses 43 and 57 (1-octen-3-ol), 41 and 93 (β-pinene), 93 and 119 (α-*trans*-bergamotene), and 93 and 121 (γ-elemene). Volatile compounds were quantified and are reported as a relative area percent by GC/FID using an Agilent 7890B and Agilent J & W DB-5, 60 m × 0.25 mm, 0.25 μm film thickness, fused silica capillary column. Operating conditions: 0.1 μL of sample (20% soln. for essential oils in ethanol, 1% for reference compounds in ethanol, 0.1% soln. for C7–C30 alkanes in hexane), 25:1 split injection, initial oven temperature at 40 °C with an initial hold time of 2 min, oven ramp rate of 3.0 °C per minute to 250 °C with a hold time of 3 min, helium carrier gas. Essential oil samples were analyzed in triplicate by GC/FID to ensure repeatability (standard deviation < 1 for all compounds). Compounds were assigned using retention indices coupled with the retention time data of reference compounds (MilliporeSigma, Sigma-Aldrich, St. Louis, MO, USA). 

The hydrogen and carbon stable isotope ratios of essential oils were analyzed by GC/IRMS using a Thermo TRACE 1310 gas chromatograph coupled to a Thermo Delta V Advantage Isotope Ratio Mass Spectrometer (ThermoFisher Scientific, Waltham, MA, USA), with an Agilent J & W DB-5, 60 m × 0.25 mm, 0.25 μm film thickness, fused silica capillary column, and a 10.0 μL syringe. 

Essential oil samples were prepared for GC/IRMS analysis in the following manner: 35 mg of sample was weighed into a 2 mL clear glass vial and brought up to 1 mL with hexane. A 100 µL aliquot was removed and placed into a second vial. The second vial was then brought up to 1 mL with hexane. This first aliquot was then used for ^2^H/^1^H analysis. From the second sample vial prepared for ^2^H/^1^H analysis, a 90 uL aliquot was removed and placed into a third vial. The resulting solution was brought to 1 mL in hexane and used for ^13^C/^12^C analysis.

GC operating conditions: splitless injection of 1 μL of sample with splitless time set at 0.25 min, injection port 270 °C, initial oven temperature of 50 °C with initial hold time of 2.0 min, oven ramp rate of 6.0 °C per minute to 250 °C with a hold time of 2.0 min, then an oven ramp rate of 10.0 °C per minute to 310 °C with a hold time of 7.0 min, helium carrier gas with constant flow 1.55 mL/min. After passing through the capillary column, samples were sent through the HTC reactor for ^2^H/^1^H analysis or the combustion reactor for ^13^C/^12^C analysis. HTC reactor temperature was set to 1410 °C and was regularly conditioned by injecting 1 µL of hexane in backflush mode. Combustion reactor temperature was set to 1000 °C. This reactor was also conditioned with oxygen at regular intervals.

To normalize IRMS results, reference materials were purchased from Dr. Arndt Schimmelmann at Indiana University and from the United States Geological Survey (USGS)—Reston Stable Isotope Laboratory. *δ*^2^H isotope ratios are expressed relative to VSMOW and *δ*^13^C isotope ratios to VPDB. The following three reference materials, along with their known values, were used to normalize results: hexadecane #C (USGS69), *δ*^2^H: 381.4‰, *δ*^13^C: −0.57‰; nonadecane #2, *δ*^2^H: −56.3‰, *δ*^13^C: −31.99‰; and tetradecanoic acid methyl ester #14M, *δ*^2^H: −231.2‰, *δ*^13^C: −29.98‰. 

Samples were analyzed in triplicate to ensure repeatability. Isotope ratios were determined for the following prominent volatile compounds: 1,8-cineole (samples D–I), *cis*-β-ocimene (samples D–I), eugenol (samples D–I), methyl eugenol (samples A–C), and (E)-caryophyllene (samples A–I). *δ*^2^H values are reported with a standard deviation < 2.8‰ and *δ*^13^C values are reported with a standard deviation < 0.2‰.

## Figures and Tables

**Table 1:** *Ocimum campechianum* essential oil profiles (*n* = 9) from Pueblo Libre (A–C), El Diamante (D–F), and Villa Rica (G–I). The Kovat’s Index (KI), volatile compound name, and compound average area % for each sample are provided. Each essential oil sample was analyzed in triplicate to ensure repeatability (standard deviation < 1 for all values). Values less than 0.1% are denoted as trace (t) and those not detected in that sample as not detectable (nd). The KI values were previously calculated by Robert Adams using a linear calculation on a DB-5 column [32].

KI	Compound	A	B	C	D	E	F	G	H	I
846	(2*E*)-hexenal	nd	nd	nd	nd	nd	nd	t	t	nd
850	(3*Z*)-hexenol	0.2	0.1	0.1	0.2	0.1	0.1	0.2	0.2	t
921	tricyclene	nd	nd	nd	t	t	0.1	0.1	t	t
924	α-thujene	nd	nd	nd	t	t	t	0.1	t	t
932	α-pinene	0.2	0.1	0.1	2.4	1.5	2.6	2.7	0.9	1.5
946	camphene	t	t	t	0.5	0.3	0.5	0.5	0.2	0.3
969	sabinene	0.1	t	t	0.9	0.6	0.9	1.0	0.4	0.7
974	1-octen-3-ol	0.1	0.1	0.1	0.1	t	t	nd	nd	nd
974	β-pinene	0.3	0.2	0.2	4.8	3.0	5.2	5.4	2.1	3.1
979	3-octanone	t	t	nd	t	t	t	t	t	t
988	myrcene	0.1	0.1	t	1.3	0.9	1.4	1.5	0.6	0.9
988	3-octanol	t	t	t	0.1	t	0.1	0.1	t	t
1001	(3*E*)-hexenyl acetate	t	nd	nd	nd	nd	nd	nd	nd	Nd
1008	*δ*-3-carene	nd	nd	nd	0.1	t	0.1	0.1	t	t
1014	α-terpinene	nd	nd	nd	0.1	t	0.1	0.1	t	t
1020	*p*-cymene	nd	t	nd	0.1	t	0.1	0.1	t	0.1
1024	limonene	0.1	0.3	0.1	1.0	0.6	1.1	1.0	0.5	0.7
1026	1,8-cineole	1.6	1.0	0.8	24.6	14.8	25.2	23.4	11.3	15.2
1032	(*Z*)-β-ocimene	1.4	1.0	0.8	15.2	9.5	15.9	18.5	7.1	12.4
1036	benzene acetaldehyde	t	t	0.1	nd	nd	nd	nd	nd	nd
1044	(*E*)-β-ocimene	0.2	0.1	0.1	1.2	0.8	1.3	1.3	0.5	1.0
1054	γ-terpinene	nd	nd	nd	0.1	t	0.1	0.1	t	t
1065	*cis*-sabinene hydrate	nd	nd	nd	t	t	t	t	t	0.1
1086	terpinolene	nd	nd	nd	0.1	t	0.1	t	t	t
1095	linalool	0.1	0.1	0.1	4.5	3.1	4.4	3.2	3.0	2.0
1140	neo-allo-ocimene	0.2	0.2	0.1	2.7	1.7	2.8	3.2	1.3	2.2
1141	camphor	nd	nd	nd	t	t	t	t	t	t
1155	isoborneol	t	t	t	0.3	0.3	0.3	0.4	0.3	0.3
1174	terpinen-4-ol	t	t	nd	t	t	t	t	t	t
1186	α-terpineol	0.1	0.1	t	0.4	0.5	0.4	0.5	0.5	0.4
1194	myrtenol	nd	nd	nd	t	t	t	t	t	t
1195	methyl chavicol	0.1	t	t	nd	nd	nd	nd	nd	nd
1239	carvone	nd	t	nd	nd	nd	nd	nd	nd	nd
1335	*δ*-elemene	0.1	0.1	0.1	0.1	0.2	0.1	0.1	0.1	0.2
^1^ 1343	bicycloelemene	1.2	1.2	1.2	1.6	2.4	1.5	1.1	2.0	2.4
1356	eugenol	1.3	1.7	1.1	15.7	30.2	15.4	17.9	38.1	26.3
1374	α-copaene	0.2	0.2	nd	t	t	t	t	t	t
^1^ 1379	unknown compound	t	t	t	0.3	0.5	0.3	0.3	0.4	0.5
1387	β-bourbonene	t	t	0.3	t	t	t	t	t	t
1389	β-elemene	3.1	3.3	3.3	4.8	6.9	4.6	3.5	5.0	7.5
1403	methyl eugenol	68.1	68.7	68.3	0.6	1.1	0.2	0.1	8.6	1.6
1409	α-gurjunene	nd	nd	nd	t	t	t	t	t	t
1417	(*E*)-caryophyllene	11.1	10.5	11.4	8.2	9.5	7.9	6.7	7.8	10.5
1432	α-*trans*-bergamotene	0.3	0.2	0.1	0.2	0.4	0.3	0.2	0.3	0.4
1434	γ-elemene	0.1	0.3	0.3	0.5	0.7	0.4	0.3	0.5	0.5
1439	aromadendrene	nd	nd	nd	0.1	0.1	0.1	t	0.1	0.1
1452	α-humulene	1.9	1.8	2.0	1.4	1.8	1.4	1.1	1.5	1.9
1458	allo-aromadendrene	0.3	0.3	0.3	0.4	0.6	0.4	0.3	0.4	0.5
1480	germacrene D	nd	nd	nd	0.2	0.2	0.1	0.1	0.2	0.2
1489	β-selinene	3.0	3.0	3.5	0.6	0.8	0.5	0.5	0.8	0.7
1500	bicyclogermacrene	3.6	3.7	4.1	2.9	4.3	2.7	2.1	3.5	4.2
1505	β-bisabolene	nd	t	nd	nd	nd	nd	nd	nd	nd
1521	*δ*-cadinene	nd	t	nd	nd	nd	nd	nd	nd	nd
1545	selina-3,7(11)-diene	t	nd	0.1	nd	nd	nd	nd	nd	nd
1559	germacrene B	t	0.1	0.1	0.1	0.1	0.1	0.1	0.1	0.1
1577	spathulenol	t	0.1	0.1	0.1	0.3	0.1	0.2	0.1	0.2
1582	caryophyllene oxide	0.1	0.2	0.2	0.2	0.5	0.2	0.3	0.2	0.3
1592	viridiflorol	t	t	nd	nd	nd	nd	nd	nd	nd
1608	humulene epoxide II	t	nd	nd	t	0.1	t	t	t	t
1618	junenol	nd	0.1	nd	nd	nd	nd	nd	nd	nd
1649	β-eudesmol	t	0.1	0.1	t	0.1	t	t	0.1	0.1
column total		99.5	99.2	99.1	99.1	98.9	99.1	98.6	99.1	99.3

^1^ KI not previously calculated [32]. Manual calculation performed using alkane standards.

**Table 2:** Stable isotope ratios, *δ*^2^H and *δ*^13^C, for 1,8-cineole, *cis*-β-ocimene, eugenol, methyl eugenol, and (*E*)-caryophyllene when compounds prominent in a sample. Values not analyzed in a sample are denoted as not analyzed (na).

Sample	1,8-cineole		*cis*-β-ocimene		eugenol		methyl eugenol		(*E*)-caryophyllene	
	*δ*^2^H (‰)	*δ*^13^C (‰)	*δ*^2^H (‰)	*δ*^13^C (‰)	*δ*^2^H (‰)	*δ*^13^C (‰)	*δ*^2^H (‰)	*δ*^13^C (‰)	*δ*^2^H (‰)	*δ*^13^C (‰)
**A**	na	na	na	na	na	na	−154.876	−31.299	−278.359	−32.688
**B**	na	na	na	na	na	na	−152.193	−31.152	−270.799	−32.800
**C**	na	na	na	na	na	na	−153.523	−32.087	−272.000	−32.850
**D**	−333.527	−31.636	−279.085	−31.171	−135.071	−31.144	na	na	−257.266	−29.102
**E**	−350.255	−32.502	−291.040	−31.208	−116.647	−31.344	na	na	−248.130	−28.913
**F**	−322.961	−31.595	−274.747	−31.093	−130.130	−31.869	na	na	−255.184	−28.956
**G**	−321.236	−32.719	−272.945	−31.724	−112.401	−32.997	na	na	−248.017	−29.838
**H**	−364.980	−33.958	−299.953	−34.969	−115.169	−33.236	na	na	−249.117	−31.612
**I**	−342.966	−31.559	−291.242	−30.309	−124.823	−31.557	na	na	−247.802	−28.059

**Table 3:** Yield data, including mass of plant material distilled (kg), essential oil yield (mL), and calculated yield (mL/kg). Average calculated yields per distillery range from 2.4–2.9 mL/kg. The relative standard deviation (RSD) is provided for essential oil yield in each region.

Distillery	Sample	Mass Distilled (kg)	Yield EO (mL)	Yield EO (mL/kg)
Pueblo Libre	A	14.5	50.0	3.4
	B	3.7	10.0	2.7
	C	3.5	9.0	2.6
	Avg.	7.2	23.0	2.9
	Avg. RSD (*n* = 3)			16.3
El Diamante	D	14.0	30.0	2.1
	E	4.0	11.0	2.8
	F	3.5	9.0	2.6
	Avg.	7.2	16.7	2.5
	Avg. RSD (*n* = 3)			12.5
Villa Rica	G	12.0	28.0	2.3
	H	4.0	11.0	2.8
	I	4.5	10.0	2.2
	Avg.	6.8	16.3	2.4
	Avg. RSD (*n* = 3)			11.4

**Table 4:** Plant collection area and associated essential oil sample names.

Plant Collection Area Name	Essential Oil Samples
Pueblo Libre	A, B, C
El Diamante	D, E, F
Villa Rica	G, H, I

## Data Availability

The data presented in this study are available upon request from the corresponding author.

## Sample Availability

Samples of the compounds are not available from the authors.
